# Association of prognostic nutritional index with all-cause and cardiovascular mortality in adults with depression: NHANES 2005–2018

**DOI:** 10.3389/fnut.2025.1599830

**Published:** 2025-05-19

**Authors:** Haiyan Ding, Xinchun Zou

**Affiliations:** ^1^Key Laboratory of Melanoma Research, Cancer Biotherapy Center, Yunnan Cancer Hospital, The Third Affiliated Hospital of Kunming Medical University, Peking University Cancer Hospital Yunnan, Kunming, China; ^2^Department of Infection Management, Kunming Medical University School and Hospital of Stomatology, Kunming, China

**Keywords:** cohort study, depression, mortality, National Health and Nutrition Examination Survey, prognostic nutritional index

## Abstract

**Introduction:**

The Prognostic Nutritional Index (PNI) reflects immune and nutritional status and has been widely used to assess various diseases. However, research on the PNI and mortality in patients with depression is limited.

**Objective:**

The study aimed to assess the association between the PNI and both all-cause and cardiovascular disease (CVD) mortality in adults with depression.

**Methods:**

This study analyzed the PNI levels in a cohort of 2,396 adults with depression. The analysis was conducted using data obtained from the National Health and Nutrition Examination Survey (NHANES), which was conducted between 2005 and 2018. Mortality outcomes were determined through a comprehensive review of the National Death Index records through December 31, 2019. Multivariable weighted Cox proportional hazards regression models were employed to investigate the hazard ratios (HRs) and 95% confidence intervals (CIs) for all-cause and CVD mortality. Restricted cubic spline analyses were utilized to explore the potential nonlinear association between PNI levels and mortality.

**Results:**

The weighted mean PNI level was 41.89 (standard error 0.1), and the median follow-up duration was 84 months. There were 295 all-cause deaths and 73 CVD deaths during the follow-up period. Higher PNI levels were significantly associated with a reduced risk of all-cause mortality (HR, 0.47; 95% confidence interval [CI], 0.31–0.73; *p* for trend < 0.001) and CVD mortality (HR, 0.51; 95% CI, 0.27–0.96; *p* for trend = 0.038) compared with lower PNI levels. Each 1-unit increase in PNI was associated with a 12% reduction in the risk of all-cause mortality (HR, 0.88; 95% CI, 0.84–0.93; *p* < 0.001) and a 12% reduction in the risk of CVD mortality (HR, 0.88; 95% CI, 0.80–0.96; *p* = 0.006). After multivariable adjustment, a linear association was observed (*p* for nonlinearity = 0.114 and 0.071 for all-cause and CVD mortality, respectively). Subgroup analyses showed that no statistically significant interactions were observed in any of the subgroups, as the *p*-values were all above 0.05.

**Conclusion:**

Higher PNI levels were associated with lower all-cause and CVD mortality in adults with depression. These findings suggest that PNI may serve as a clinically useful indicator to predict the prognosis of patients with depression.

## 1 Introduction

Depression is a prevalent mental health disorder, affecting over 300 million individuals worldwide, according to the World Health Organization (WHO) (1). The condition is characterized by persistent negative emotions, decreased interest, inability to concentrate, and significant disruptions in sleep and appetite patterns (2). Depression has been identified as a significant public health concern on a global scale, not only due to its profound impact on psychosocial functioning but also its substantial effect on quality of life (3). From 1990 to 2019, the burden of disability-adjusted life-years (DALYs) caused by depressive disorders increased by 61.1% (4). According to the National Institute of Mental Health, approximately 20.6 million U.S. residents, constituting approximately 8.4% of the adult population, experienced at least one major depressive episode in 2019. According to the 2008 World Health Organization (WHO) report, major depressive disorder (MDD) was ranked as the third leading cause of global burden of disease. Projections indicate that MDD will become the leading cause by the year 2030 (5). In addition, depression has been found to be strongly associated with several chronic diseases, such as cardiovascular disease (6), diabetes (7), and hypertensive (8). This association contributes to an increase in the overall burden of disease and mortality risk (9). A meta-analysis reveals a significant association between an inflammatory diet and the risk of depression (10). Therefore, it is imperative to investigate the prevention of depression and the improvement of its prognosis from the perspective of inflammation and nutrition.

Nutritional Psychiatry has emerged as a promising and emerging approach, providing robust evidence supporting both preventive and clinical strategies to manage neuropsychiatric disorders through dietary and nutritional interventions (11). For instance, interventions based on the Mediterranean diet have been shown to reduce depression (12). Moreover, “anti-inflammatory” dietary patterns are also associated with a reduced incidence of depression (13). In recent years, PNI has received increasing attention from the scientific community. The PNI integrates inflammatory and nutritional components and is calculated using serum albumin and total lymphocyte count (14). The initial development of the PNI by Onodera et al. was for the purpose of assessing the nutritional status of patients undergoing gastrointestinal surgery (15). Subsequent research has demonstrated the ability of PNI to serve as a prognostic indicator for patients diagnosed with various types of cancer, including non-small cell lung cancer (16), cervical cancer (17), prostate cancer (18), and oral cancer (19). In addition, PNI has been associated with adverse clinical outcomes in several diseases, including acute kidney injury (AKI) (20), rheumatoid arthritis (21), coronary artery disease (22), and migraine (23). However, research on the relationship between PNI and mortality risk in depressed adults in the U.S. is limited, suggesting a need for further investigation.

In this study, we used a long-term follow-up cohort from the National Health and Nutrition Examination Survey (NHANES) to assess the association between PNI and all-cause and CVD mortality in adults with depression.

## 2 Materials and methods

### 2.1 Study population

Data for this cohort study were obtained from the NHANES, which was conducted from 2005 to 2018. NHANES uses a multistage, stratified probability design to ensure the sample is representative of the US population (24). Ethical approval for data collection was obtained from the Ethics Review Committee of the National Center for Health Statistics, and all participants provided informed consent. A total of 39,749 individuals aged 20 years or older were initially enrolled in the study. Participants were excluded based on the following criteria: loss to follow-up (*n* = 1,656), missing PHQ-9 scores or PHQ-9 scores below 10 (*n* = 35,040), missing serum albumin data (*n* = 167), missing lymphocyte counts (*n* = 22), and incomplete covariate data (*n* = 468) including variables such as sex, age, race, education level, marital status, family income, drinking status, smoking status, BMI, stroke, coronary heart disease, hypertension, and diabetes. The final analytic cohort consisted of 2,396 individuals with depression. The study was conducted in accordance with the Strengthening the Reporting of Observational Studies in Epidemiology (STROBE) reporting guidelines.

### 2.2 Calculation of PNI

The PNI was calculated using the following formula: PNI=10Xalbumin(g/dL)+0.005Xabsolute lymphocyte count(10^3cells/μL). Lymphocyte count was determined by complete blood cell count (CBC) tests, with CBC parameters measured using the Beckman Coulter counting and sizing method. Serum albumin (ALB) levels were analyzed using an automated chemistry analyzer, the Beckman Synchron LX20.

### 2.3 Assessment of depression

The analyses were based on data collected from participants during the seven 2-year cycle of NHANES (2005–2018). The Patient Health Questionnaire (PHQ-9) is a 9-item screening tool that assesses the severity of depressive symptoms experienced by patients during the past 2 weeks. The instrument has been validated as a reliable diagnostic tool for depression and is consistent with the DSM-IV criteria for depression (25). Each PHQ-9 item is scored 0–3, with total scores ranging from 0 to 27. Participants were classified as having depression if their PHQ-9 score was 10 or above, and as not having depression if their score was below 10 (26, 27). The threshold has been demonstrated and validated, with a sensitivity and specificity of 88%, respectively, for diagnoses of depressive disorder (25).

### 2.4 Assessment of mortality

Mortality data were obtained by linking the cohort database to the Centers for Disease Control and Prevention (CDC) National Death Index as of December 31, 2019. All-cause mortality was defined as death from any cause. The definition of CVD mortality was derived from the International Classification of Diseases, Tenth Revision (ICD-10) and includes the following disease codes: I00-I09 (acute rheumatic fever and chronic rheumatic heart diseases), I11 (hypertensive heart disease), I13 (hypertensive heart and renal disease), I20-I25 (ischemic heart diseases), I26-I28 (pulmonary embolism and other acute pulmonary heart diseases), I29 (various cardiovascular diseases caused by different reasons), I30-I51 (other forms of heart disease), and I60-I69 (cerebrovascular diseases) (23).

### 2.5 Assessment of covariates

In accordance with existing research, the following covariates were included: age, sex, marital status, race, education level, family income, smoking status, drinking status, body mass index (BMI), stroke, coronary heart disease, hypertension and diabetes. Marital status was categorized into three groups: married or living with a partner and living alone. Race categories included Non-Hispanic White, Non-Hispanic Black, Mexican American, and others. Participants’ education level was divided into three categories: less than 9 years, 9–12 years, and more than 12 years. Family income was categorized into three levels: ≤1.30, 1.31–3.50, and >3.50 (28). Smoking status was categorized as follows: never, current, or former. Drinking status included the following categories: never drinkers (<12 drinks in their lifetime), former drinkers (≥ 12 drinks in 1 year and did not drink last year, or did not drink last year but drank ≥ 12 drinks in their lifetime), and current drinkers (≥12 drinks in any 1 year and did drink last year) (23). BMI was categorized into three groups: less than 25 kg/m^2^, 25 to less than 30 kg/m^2^, and 30 kg/m^2^ or above. The diagnosis of diabetes was based on the following criteria: a doctor’s diagnosis, glycohemoglobin HbA1c ≥ 6.5 (%), Fasting Plasma Glucose (FPG) ≥ 7.0 (mmol/L), a random plasma glucose ≥ 11.1 (mmol/L), 2-h plasma glucose from an oral glucose tolerance test (OGTT) ≥ 11.1 (mmol/L), or the use of diabetes medication or insulin (29). The diagnosis of hypertension was determined by mean blood pressure, excluding diastolic readings of zero unless all diastolic readings were zero. In the case of a single reading, it was considered the average. In the case of multiple readings, the first reading was excluded from the calculation (30). Hypertension was diagnosed if the systolic blood pressure was ≥ 140 mmHg or the diastolic blood pressure was ≥ 90 mmHg. Data on stroke and coronary heart disease were self-reported.

### 2.6 Statistical analysis

The present analysis used the complex sampling design with the application of sampling weights according to the NHANES analysis guidelines (31). Data for this study were obtained from the Home Interview and the Mobile Examination Center (MEC) components of the NHANES survey. For the period 2005–2018, the sampling weights were calculated as 1/7 × 2-year MEC weight. Follow-up times were calculated from the completion of the MEC, and the National Death Index is updated every 4 years; the most recent follow-up data are currently available as of December 31, 2019. Continuous variables are presented as mean [standard error (SE)], and categorical variables are presented as unweighted number (weighted percentage). The Wilcoxon rank-sum test, adapted for complex survey samples, was used to assess differences in continuous variables. The Chi-squared tests with Rao & Scott’s second order correction were used to analyze categorical variables.

Multivariable weighted Cox proportional hazards regression models were used to determine the hazard ratio (HR) and 95% confidence interval (95% CI) for the association between PNI and the risks of all-cause and CVD mortality through three progressively adjusted models. Model 1 was adjusted for age, sex; Model 2 was further adjusted for race, marital status, family income, education level, smoking status, drinking status; Model 3 was further adjusted for BMI, stroke, coronary heart disease, hypertension, diabetes, NHANES cycles. Furthermore, we employed multivariate adjusted restricted cubic spline (RCS) regression to model the dose–response relationship between the PNI and all-cause and CVD mortality. PNI was treated as a continuous variable, with three knots positioned at the 10th, 50th, and 90th percentiles of its distribution. The model was adjusted for covariates as specified in model 3.

Subgroup analyses and interaction tests were performed for age (<65 vs. ≥65 years), sex (male vs. female), race (Non-Hispanic White vs. other), family income (≤1.3, 1.31–3.50, >3.5), smoking status (never, former, current), drinking status (never, former, current), BMI (<25, ≥25 to < 30, ≥30 kg/m^2^), coronary heart disease (no vs. yes), hypertension (no vs. yes), and diabetes (no vs. yes). These analyses were performed using multivariable Cox proportional hazards regression models, adjusting for the same covariates as in model 3 except for the stratification factor itself. The likelihood ratio tests assessed subgroup interactions, and the results were displayed in a forest plot.

In addition, several sensitivity analyses were conducted to assess the robustness of the research findings. First, multiple imputation procedures were used for individuals with missing covariate data, based on five replications. Second, participants with a history of cancer were excluded from the analysis, given the potential for cancer to increase mortality risk.

All analyses were performed using R statistical software (version 4.2.2, http://www.R-project.org, The R Foundation) and the Free Statistics analysis platform (version 2.0, Beijing, China). Statistical significance was defined as a two-sided *p*-value less than 0.05. Data analysis was conducted between February and March 2025.

## 3 Results

### 3.1 Study population

**Figure 1:**
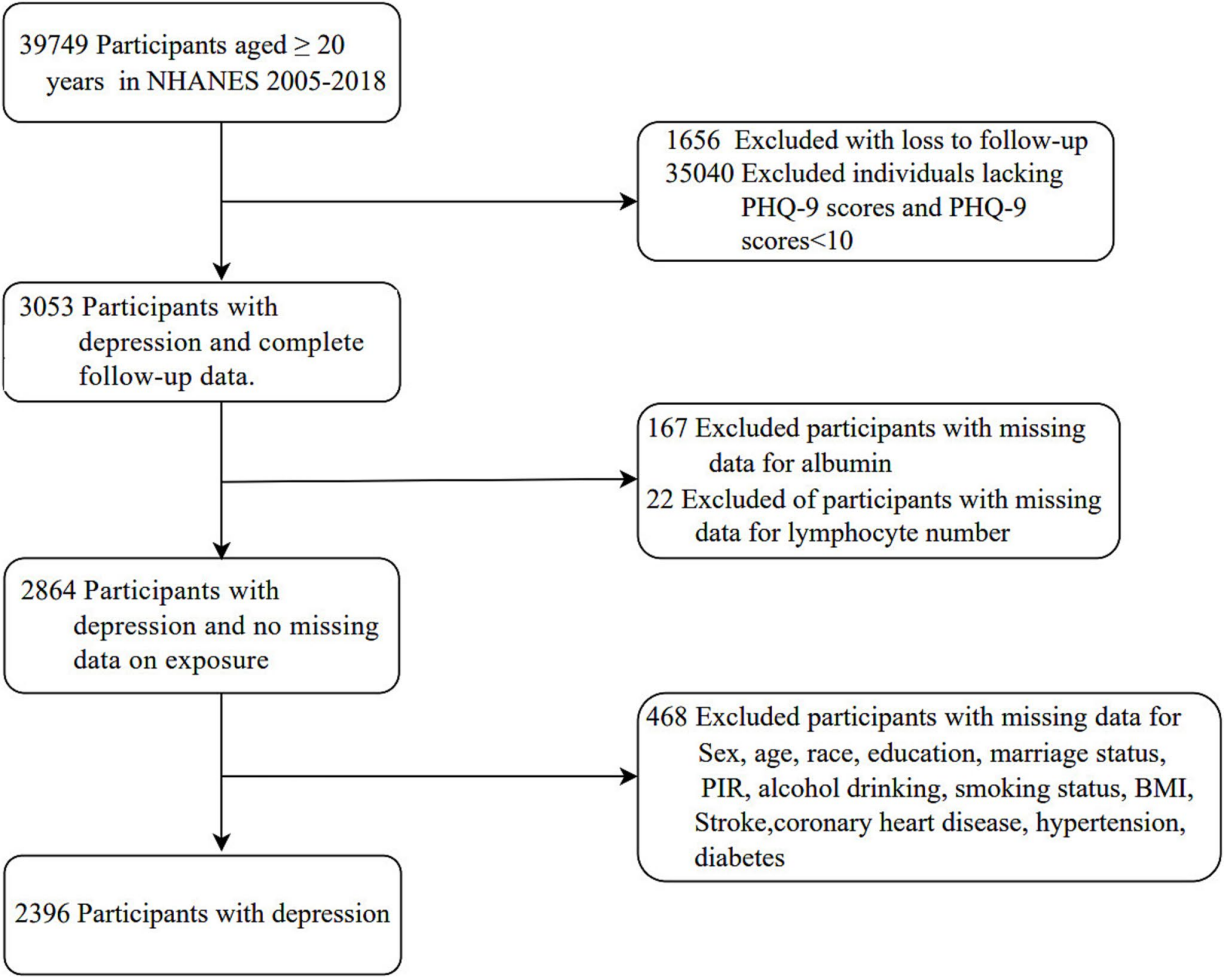
Flow chart of the study.

Of a total of 39,749 participants aged ≥ 20 years who completed interviews and underwent MEC screening, 37,353 were excluded based on the following criteria: loss to follow-up (*n* = 1,656), missing PHQ-9 scores and PHQ-9 scores below 10 (*n* = 35,040), missing data on exposures (*n* = 189), and missing data on covariates (*n* = 468). Consequently, the analysis was performed on a sample of 2,396 participants (Figure 1).

### 3.2 Baseline characteristics

In the cohort of 2,396 adults with depression (mean age 46.42 years; 63.70% female), the mean PNI was 41.89 (SE, 0.1). Table 1 presents the baseline characteristics of the study population categorized by PNI. Participants with higher PNI levels were more likely to be younger, female, non-Hispanic White, and have a lower BMI. These participants also had a lower prevalence of coronary heart disease, hypertension, and diabetes.

**Table 1:** Characteristics of participants in the NHANES 2005–2018.

Characteristic	PNI				*p*-value
	Total	Tertile 1 (<40)	Tertile 2 (≥40, <43)	Tertile 3 (≥43)	
No.	2,396	794	795	807	
Age (years), mean (SE)	46.42 (0.45)	49.63 (0.69)	46.71 (0.62)	43.53 (0.76)	<0.001
Sex, *n* (%)					<0.001
Male	883 (36.30%)	217 (26.15%)	270 (32.54%)	396 (47.83%)	
Female	1,513 (63.70%)	577 (73.85%)	525 (67.46%)	411 (52.17%)	
Race, *n* (%)					<0.001
Non-Hispanic White	1,066 (65.62%)	323 (62.56%)	332 (64.06%)	411 (69.47%)	
Non-Hispanic Black	510 (12.73%)	227 (18.48%)	163 (12.59%)	120 (8.14%)	
Mexican American	350 (7.56%)	106 (7.03%)	136 (8.93%)	108 (6.84%)	
Others	470 (14.08%)	138 (11.93%)	164 (14.42%)	168 (15.55%)	
Marital status, *n* (%)					0.68
Married or living with a partner	1,082 (48.33%)	346 (48.34%)	370 (46.74%)	366 (49.67%)	
Living alone	1,314 (51.67%)	448 (51.66%)	425 (53.26%)	441 (50.33%)	
Family income, *n* (%)					0.15
≤ 1.30	1,249 (40.68%)	439 (44.49%)	377 (36.56%)	433 (41.03%)	
1.31–3.50	803 (36.37%)	245 (31.86%)	295 (40.25%)	263 (36.79%)	
> 3.50	344 (22.96%)	110 (23.65%)	123 (23.20%)	111 (22.18%)	
Education level (year), *n* (%)					0.19
<9	324 (7.72%)	102 (7.55%)	116 (8.64%)	106 (7.09%)	
9–12	1,071 (43.39%)	378 (47.24%)	352 (42.90%)	341 (40.64%)	
>12	1,001 (48.89%)	314 (45.21%)	327 (48.46%)	360 (52.27%)	
Smoking status, *n* (%)					0.15
Never	951 (37.96%)	324 (38.73%)	324 (37.79%)	303 (37.47%)	
Former	545 (22.66%)	199 (26.28%)	178 (21.27%)	168 (20.87%)	
Current	900 (39.38%)	271 (34.99%)	293 (40.95%)	336 (41.66%)	
Drinking status, *n* (%)					0.19
Never	286 (8.78%)	111 (11.43%)	103 (7.87%)	72 (7.37%)	
Former	522 (19.43%)	181 (19.50%)	169 (19.29%)	172 (19.48%)	
Current	1,588 (71.79%)	502 (69.07%)	523 (72.84%)	563 (73.14%)	
BMI (kg/m^2^), *n* (%)					<0.001
<25	571 (25.95%)	104 (14.89%)	191 (26.14%)	276 (34.87%)	
≥25, <30	629 (26.18%)	171 (19.48%)	215 (27.46%)	243 (30.60%)	
≥30	1,196 (47.87%)	519 (65.63%)	389 (46.41%)	288 (34.53%)	
Coronary heart disease, *n* (%)					0.002
No	2,248 (94.52%)	734 (91.32%)	750 (95.89%)	764 (96.00%)	
Yes	148 (5.48%)	60 (8.68%)	45 (4.11%)	43 (4.00%)	
Stroke, *n* (%)					0.13
No	2,209 (93.58%)	719 (92.02%)	734 (93.33%)	756 (95.07%)	
Yes	187 (6.42%)	75 (7.98%)	61 (6.67%)	51 (4.93%)	
Hypertension, *n* (%)					<0.001
No	1,188 (52.92%)	332 (44.30%)	401 (53.47%)	455 (59.53%)	
Yes	1,208 (47.08%)	462 (55.70%)	394 (46.53%)	352 (40.47%)	
Diabetes, *n* (%)					<0.001
No	1,777 (79.91%)	507 (69.77%)	606 (81.46%)	664 (86.94%)	
Yes	619 (20.09%)	287 (30.23%)	189 (18.54%)	143 (13.06%)	
PNI, mean (SE)	41.89 (0.10)	37.92 (0.09)	41.60 (0.04)	45.39 (0.08)	<0.001

Data are presented as mean (standard error) for continuous variables and unweighted number (weighted percentage) for categorical variables. BMI, body mass index; PNI, prognostic nutritional index; NHANES, National Health and Nutrition Examination Survey.

### 3.3 PNI and mortality

A total of 295 all-cause deaths and 73 CVD deaths were identified during a median follow-up of 84 months. Following multivariable adjustment for confounders, including age, sex, race, marital status, family income, education level, smoking status, drinking status, BMI, stroke, coronary heart disease, hypertension, diabetes, and NHANES cycle, the relationship between PNI and mortality risk was evaluated. The analyses showed that higher PNI levels were significantly associated with reduced risk of all-cause mortality (HR, 0.47; 95% CI, 0.31–0.73; *p* for trend < 0.001) and CVD mortality (HR, 0.51; 95% CI, 0.27–0.96; *p* for trend = 0.038) compared with lower PNI levels (Table 2).

**Table 2:** Hazard ratios of all-cause and CVD mortality among adults with depression in NHANES 2005–2018.

Variables	Deaths, no./Total, no.	Crude model		Model 1		Model 2		Model 3	
		HR (95% CI)	*p*-value	HR (95% CI)	*p*-value	HR (95% CI)	*p*-value	HR (95% CI)	*p*-value
All-cause mortality									
PNI	295/2396	0.87 (0.84, 0.91)	<0.001	0.90 (0.85, 0.94)	<0.001	0.89 (0.85, 0.94)	<0.001	0.88 (0.84, 0.93)	<0.001
Tertile 1 (<40)	138/794	Reference		Reference		Reference		Reference	
Tertile 2 (≥40, <43)	84/795	0.54 (0.37, 0.78)	0.001	0.67 (0.46, 0.98)	0.038	0.68 (0.46, 0.99)	0.044	0.63 (0.43, 0.94)	0.022
Tertile 3 (≥43)	73/807	0.39 (0.26, 0.57)	<0.001	0.54 (0.36, 0.82)	0.004	0.52 (0.34, 0.81)	0.004	0.47 (0.31, 0.73)	<0.001
*P* for trend			<0.001		0.003		0.003		<0.001
CVD mortality									
PNI	73/2396	0.86 (0.80, 0.93)	<0.001	0.89 (0.82, 0.98)	0.011	0.89 (0.81, 0.98)	0.018	0.88 (0.80, 0.96)	0.006
Tertile 1 (<40)	38/794	Reference		Reference		Reference		Reference	
Tertile 2 (≥40, <43)	13/795	0.27 (0.12, 0.58)	<0.001	0.38 (0.17, 0.83)	0.015	0.37 (0.17, 0.83)	0.016	0.34 (0.16, 0.71)	0.004
Tertile 3 (≥43)	22/807	0.39 (0.21, 0.72)	0.003	0.62 (0.32, 1.18)	0.147	0.56 (0.28, 1.10)	0.092	0.51 (0.27, 0.96)	0.038
*P* for trend			0.005		0.132		0.082		0.038

Model 1, Adjusted for age, sex; Model 2, Further adjusted for race, marital status, family income, education level, smoking status, drinking status; Model 3, Further adjusted for BMI, stroke, coronary heart disease, hypertension, diabetes, NHANES cycle. CVD, cardiovascular disease; BMI, body mass index; PNI, prognostic nutritional index; NHANES, National Health and Nutrition Examination Survey; HR, hazard ratio; CI, confidence interval.

**Figure 2:**
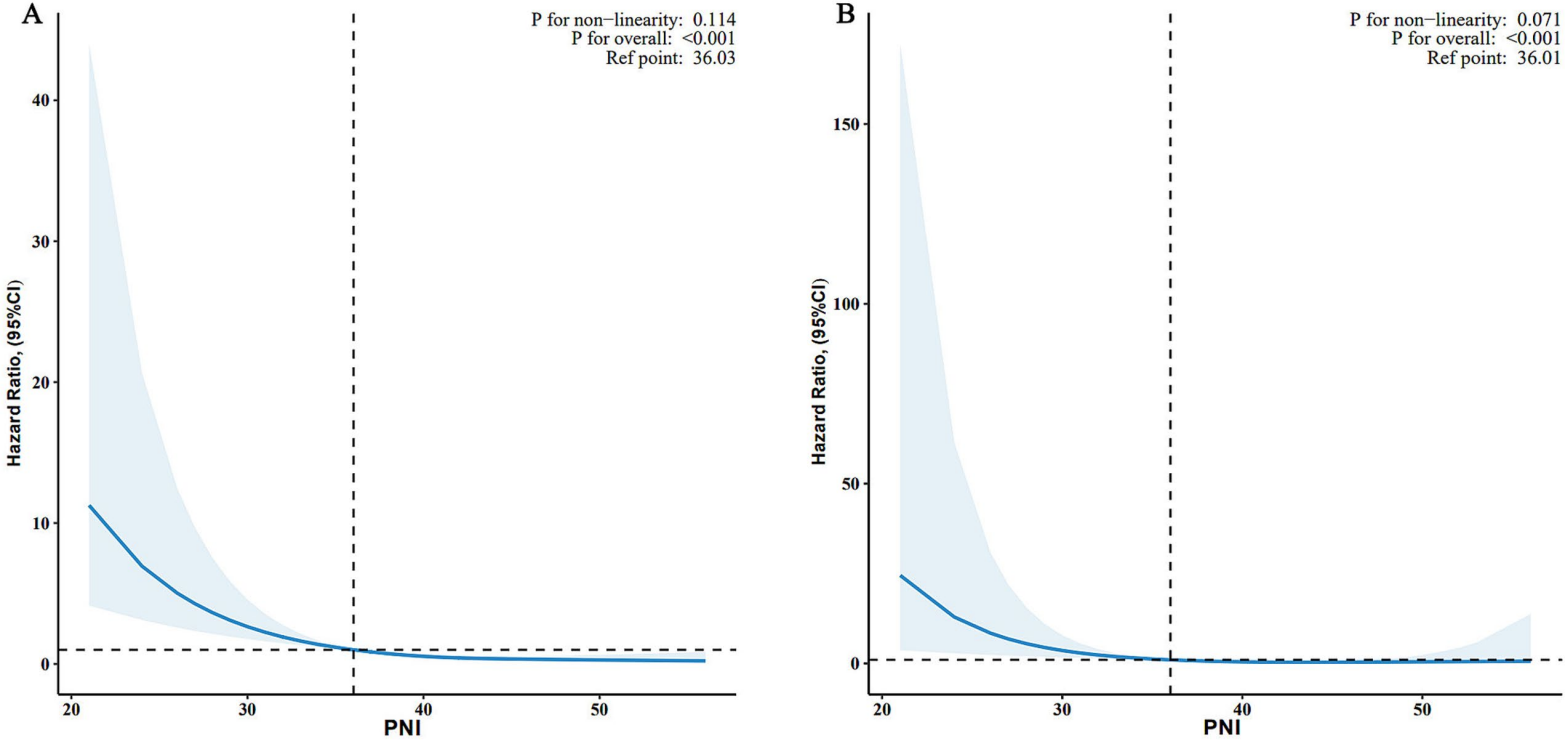
Associations between PNI and all-cause **(A)** and CVD mortality **(B)** among adults with depression in NHANES 2005–2018. HRs were adjusted for age, sex, race, marital status, family income, education level, smoking status, drinking status, BMI, stroke, coronary heart disease, hypertension, diabetes, NHANES cycle. PNI, prognostic nutritional index; CVD, cardiovascular disease; BMI, body mass index; HR, hazard ratio; CI, confidence interval; NHANES, National Health and Nutrition Examination Survey; RCS, restricted cubic spline.

As shown in Figure 2, the relationship between PNI and all-cause mortality (A) and CVD mortality (B) was illustrated by a dose–response curve. After multivariable adjustment, a linear relationship was observed (*p* for nonlinearity = 0.114 and 0.071 for all-cause and CVD mortality, respectively). When PNI was analyzed as a continuous variable, each 1-unit increase in PNI was associated with a 12% lower risk of all-cause mortality (HR, 0.88; 95% CI, 0.84–0.93; *p* < 0.001) and a 12% lower risk of CVD mortality (HR, 0.88; 95% CI, 0.80–0.96; *p* = 0.006) (Table 2).

### 3.4 Subgroup analyses

**Figure 3:**
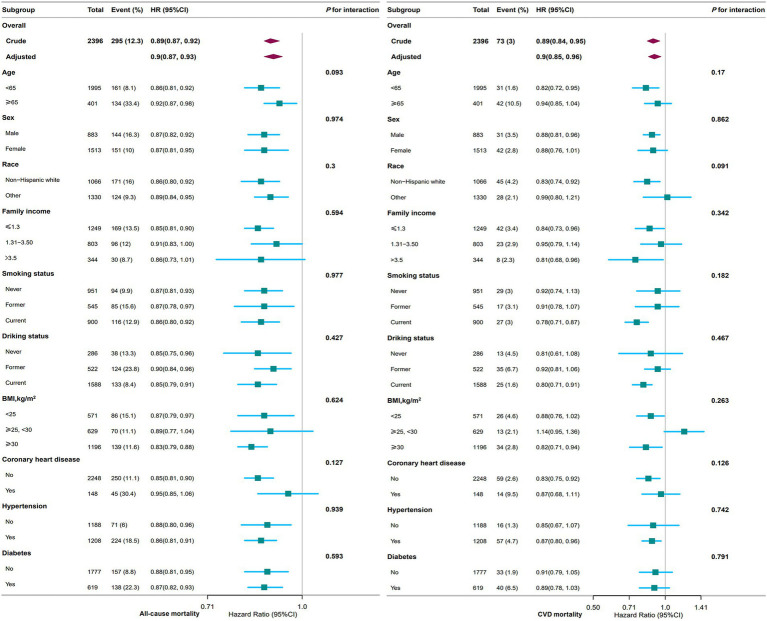
Association between PNI and all-cause and CVD mortality among adults with depression in NHANES 2005–2018. The stratifications were adjusted for all variables (age, sex, race, marital status, family income, education level, smoking status, drinking status, BMI, stroke, coronary heart disease, hypertension, diabetes, NHANES cycle) except for the stratification factor itself. Squares represent the HRs and horizontal lines represent 95% CIs. Diamonds represent the overall HR, and the outer points of the diamonds represent the 95% CI. PNI, prognostic nutritional index; CVD, cardiovascular disease; BMI, body mass index; HR, hazard ratio; CI, confidence interval; NHANES, National Health and Nutrition Examination Survey.

Figure 3 shows subgroup analyses of the associations between PNI and all-cause and CVD mortality. With respect to all-cause mortality, elevated PNI levels were associated with a reduced risk in specific subgroups: participants aged < 65 years (HR, 0.86; 95% CI, 0.81–0.92), non-Hispanic White (HR, 0.86; 95% CI, 0.80–0.92), individuals with family income ≤1.3 (HR, 0.85; 95% CI, 0.81–0.90), individuals with BMI ≥ 30 kg/m^2^ (HR, 0.83; 95% CI, 0.79–0.88), participants without coronary heart disease (HR, 0.85; 95% CI, 0.81–0.90), and those with hypertension (HR, 0.86; 95% CI, 0.81–0.91).

For the analysis of CVD mortality, elevated PNI levels were associated with a reduced risk in the following subgroups: participants aged < 65 years (HR, 0.82; 95% CI, 0.72–0.95), non-Hispanic White (HR, 0.83; 95% CI, 0.74–0.92), those with family income > 3.5 (HR, 0.81; 95% CI, 0.68–0.96), current smokers (HR, 0.78; 95% CI, 0.71–0.87), current drinkers (HR, 0.80; 95% CI, 0.71–0.91), individuals with BMI ≥ 30 kg/m^2^ (HR, 0.82; 95% CI, 0.71–0.94), participants without coronary heart disease (HR, 0.83; 95% CI, 0.75–0.92).

Notably, no statistically significant interactions were observed in any of the subgroup analyses (all *p* > 0.05).

### 3.5 Sensitivity analysis

The results of sensitivity analyses are summarized in Table 3. We performed multiple imputations of missing covariates for the remaining 2,864 participants. The analysis showed that participants with higher PNI levels were associated with a 51% lower risk of all-cause mortality compared to those with lower PNI levels (adjusted HR, 0.49; 95% CI, 0.32–0.74; *p* < 0.001). To account for potential confounding by pre-existing conditions, we excluded 270 participants with a history of cancer at baseline (*n* = 2,126). Despite excluding these participants, the association remained statistically significant, with an adjusted HR of 0.37 (95% CI, 0.22–0.64; *p* < 0.001).

**Table 3:** Sensitivity analyses.

Variables	Deaths, no./Total, no.	Model 1		Model 2		Model 3	
		HR (95% CI)	*p*-value	HR (95% CI)	*p*-value	HR (95% CI)	*p*-value
Multiple imputation of missing data							
Tertile 1 (<40)	162/949	Reference		Reference		Reference	
Tertile 2 (≥40, <43)	108/945	0.66 (0.48, 0.92)	0.014	0.65 (0.47, 0.92)	0.013	0.66 (0.47, 0.92)	0.014
Tertile 3 (≥43)	84/970	0.49 (0.33, 0.73)	<0.001	0.48 (0.31, 0.72)	<0.001	0.49 (0.32, 0.74)	<0.001
*p* for trend			<0.001		<0.001		<0.001
Excluding participants who had a history of cancer at baseline							
Tertile 1 (<40)	114/695	Reference		Reference		Reference	
Tertile 2 (≥40, <43)	63/709	0.62 (0.40, 0.94)	0.025	0.59 (0.39, 0.91)	0.016	0.57 (0.37, 0.88)	0.011
Tertile 3 (≥43)	48/722	0.45 (0.26, 0.76)	0.003	0.42 (0.24, 0.71)	0.001	0.37 (0.22, 0.64)	<0.001
*p* for trend			0.002		<0.001		<0.001

Model 1, Adjusted for age, sex; Model 2, Further adjusted for race, marital status, family income, education level, smoking status, drinking status; Model 3, Further adjusted for BMI, stroke, coronary heart disease, hypertension, diabetes, NHANES cycle. BMI, body mass index; NHANES, National Health and Nutrition Examination Survey; HR, hazard ratio; CI, confidence interval.

## 4 Discussion

The results of this prospective cohort study of adults with depression in the United States showed a significant inverse association between the PNI and both all-cause and CVD mortality. The association remained consistent after adjustment for conventional risk factors. These risk factors included age, sex, race, marital status, family income, education level, smoking status, drinking status, BMI, stroke, coronary heart disease, hypertension, diabetes, and NHANES cycle. The robustness of this association was further confirmed by sensitivity and stratified analyses, suggesting that higher PNI levels may serve as a protective factor against mortality risk in this population.

The PNI, calculated from serum albumin and lymphocyte count, is a well-established marker of nutritional and immunological status. As shown in previous studies, its prognostic utility has been demonstrated in several cancers and chronic diseases. For instance, in colorectal cancer (CRC) (32), an increase in each standard deviation (SD) increase in PNI was associated with a 2.3% reduction in progression-free survival (PFS: HR, 0.977; 95% CI, 0.962–0.993; *p* = 0.004) and overall survival (OS: HR, 0.977; 95% CI, 0.962–0.993; *p* = 0.004). In addition, in patients with advanced lung cancer patients receiving immunotherapy, elevated PNI levels showed a significant correlation with improved survival outcomes (OS: HR, 2.56; 95% CI, 1.86–3.54; PFS: HR, 1.91; 95% CI, 1.53–2.40) and improved response to anti-cancer therapy (33). In addition to its applications in oncology, PNI has been independently associated with all-cause and CVD mortality in individuals with heart failure (34), diabetes or prediabetes (35), non-alcoholic fatty liver disease (36), frailty (37), and chronic kidney disease (38).

A number of hypotheses have been proposed to explain the mechanisms underlying the relationship between PNI and depression. In particular, PNI serves as a comprehensive marker reflecting both chronic inflammatory status and nutritional status (39). Serum albumin, a key component of the PNI, has several beneficial physiological properties, such as stabilizing oxidative stress reactions and regulating inflammatory responses (40). In particular, hypoalbuminemia has the potential to serve as a predictive biomarker for inflammation (41). Another critical component of the PNI is a low lymphocyte count, which serves as a marker of immune dysregulation and is strongly associated with systemic inflammatory responses (42). In cases of depression, the disorder has been observed to directly activate the hypothalamic–pituitary–adrenal (HPA) axis. This activation has been shown to result in a disruption of immune regulation, manifested by changes in immune cell populations and altered cytokines levels (43). This mechanism is supported by the consistent systemic inflammatory responses exhibited by patients with major depressive disorder (MDD) compared to healthy controls, regardless of comorbidities. The hallmarks of inflammation include elevated levels of pro-inflammatory cytokines and their soluble receptors in both peripheral blood and cerebrospinal fluid (CSF), along with increased concentrations of acute-phase proteins, chemokines, adhesion molecules, and inflammatory mediators such as prostaglandins (44, 45).

Research in nutritional psychiatry lends further support to the notion that healthy dietary patterns and specific components with anti-inflammatory effects may benefit mental health. For instance, high intakes levels of fruits, vegetables, fish, and whole grains may be associated with a reduced risk of depression (46). Meta-analyses of longitudinal studies indicated that individuals with more inflammatory dietary patterns have an increased risk of developing depression (47). Current studies suggest that diet is the essential factor for human gut microbiota composition. A plant-based diet may be an effective way to promote a diverse ecosystem of beneficial microbes that support overall health (48). Intervention studies have demonstrated that adherence to the Mediterranean diet can significantly reduce the incidence of CVD (49). Moreover, evidence from animal models suggests that diets high in fat and sucrose, characteristic of the Western diet, can impair neurogenesis and adversely impact cognitive performance (50). The influence of diet on mental health may be exerted through various biological mechanisms, including inflammation, oxidative stress, epigenetics, mitochondrial dysfunction, gut microbiota, tryptophan-kynurenine metabolism, the HPA axis, neurogenesis, brain-derived neurotrophic factor (BDNF), and obesity (51).

In summary, the relationship between PNI and depression is complex. PNI has been shown to reflect nutrition, immune function, and chronic inflammation. Depression may lower PNI by affecting the HPA axis and the immune system. Conversely, low PNI resulting from inflammation or poor nutrition has been shown to contribute to the development of depression. This bidirectional relationship demonstrates how these conditions can exacerbate each other and highlights their interrelated mechanisms.

This study has several strengths. First, it is a comprehensive prospective cohort study with a large sample size, which increases the reliability of the findings. Second, we rigorously adjusted for a comprehensive set of conventional risk factors was rigorously adjusted to reduce the potential for confounding. Third, the robustness of the observed association was confirmed by several sensitivity and stratified analyses. It should be noted that there are several limitations. First, the observational nature of the study precludes the establishment of a causal relationship between PNI and mortality. Second, PNI was measured at a single time point, which may not fully capture long-term fluctuations in nutritional and immune status. Third, the study included only individuals aged ≥ 20 years from the United States, which may limit the generalizability of the findings to other populations. Fourth, the baseline information of participants may have change over time, potentially confounding the true association between PNI and all-cause mortality. Finally, it is important to note that the generalizability of the findings may be limited to the specific population studied, and further research is needed in diverse settings.

## 5 Conclusion

Our research indicates a significant inverse association between elevated PNI levels and reduced risk of all-cause and CVD mortality in adults with depression. We recommend the use of PNI as a predictive tool for assessing mortality risk in this population. In addition, future studies are needed to investigate how improving nutrition and reducing inflammation might reduce mortality risk and prevent progression of depression.

## Data Availability

The datasets presented in this article are not readily available because in this study, we analyzed publicly available datasets. The National Health and Nutrition Examination Survey (NHANES) data are publicly available and can be downloaded directly. Requests to access the datasets should be directed to the NHANES website (https://www.cdc.gov/nchs/nhanes/index.htm).
